# Salicylic Acid in Otorrhœa

**Published:** 1876-02

**Authors:** 


Salicylic Acid in Otorrhœa (*Le Mouvement Medical*, November 20, 1875).—M. Van Stock has seen cases of chronic otorrhœa, which had resisted all other topical agents, yield as if by magic on the employment of salicylic acid. He is inclined to attribute this success to the anti-fermentative properties of the acid, which is more active than carbolic acid, without possessing the disagreeable and penetrating odor of the latter; neither does it interfere with the granulation and cicatrization of wounds. He uses a four per cent. solution, and recommends it particularly for use by the patient in those cases which can only be visited occasionally.

